# Identification of early and late flowering time candidate genes in endodormant and ecodormant almond flower buds

**DOI:** 10.1093/treephys/tpaa151

**Published:** 2020-11-16

**Authors:** Ángela S Prudencio, Frank A Hoeberichts, Federico Dicenta, Pedro Martínez-Gómez, Raquel Sánchez-Pérez

**Affiliations:** Department of Plant Breeding, Fruit Breeding Group, CEBAS-CSIC, PO Box 164, 30100 Espinardo, Murcia, Spain; Keygene N.V., Agro Business Park 90, 6708 PW Wageningen, The Netherlands; Department of Plant Breeding, Fruit Breeding Group, CEBAS-CSIC, PO Box 164, 30100 Espinardo, Murcia, Spain; Department of Plant Breeding, Fruit Breeding Group, CEBAS-CSIC, PO Box 164, 30100 Espinardo, Murcia, Spain; Department of Plant Breeding, Fruit Breeding Group, CEBAS-CSIC, PO Box 164, 30100 Espinardo, Murcia, Spain

**Keywords:** climate change, *DAM* genes, dormancy, *Prunus dulcis*, RNA-seq, tree breeding

## Abstract

Flower bud dormancy in temperate fruit tree species, such as almond [*Prunus dulcis* (Mill.) D.A. Webb], is a survival mechanism that ensures that flowering will occur under suitable weather conditions for successful flower development, pollination and fruit set. Dormancy is divided into three sequential phases: paradormancy, endodormancy and ecodormancy. During the winter, buds need cultivar-specific chilling requirements (CRs) to overcome endodormancy and heat requirements to activate the machinery to flower in the ecodormancy phase. One of the main factors that enables the transition from endodormancy to ecodormancy is transcriptome reprogramming. In this work, we therefore monitored three almond cultivars with different CRs and flowering times by RNA sequencing during the endodormancy release of flower buds and validated the data by quantitative real-time PCR in two consecutive seasons. We were thus able to identify early and late flowering time candidate genes in endodormant and ecodormant almond flower buds associated with metabolic switches, transmembrane transport, cell wall remodeling, phytohormone signaling and pollen development. These candidate genes were indeed involved in the overcoming of the endodormancy in almond. This information may be used for the development of dormancy molecular markers, increasing the efficiency of temperate fruit tree breeding programs in a climate-change context.

## Introduction

In almond [*Prunus dulcis* (Mill.) D.A. Webb], kernels are the main commercial component (Gradziel and Martínez-Gómez 2013), and they can be consumed fresh, processed or as a functional food (Musa-Veloso et al. 2016). Together with California and Australia, the Mediterranean basin is one of most productive almond areas (FAO 2017). Almond breeding programs are focused on obtaining new cultivars that are more productive, have a higher fruit quality and are adapted to different environmental conditions. Flowering time (Martinez-Gomez et al. 2017), self-compatibility (Sánchez-Pérez et al. 2004) and kernel sweetness (Sánchez-Pérez et al. 2019) are the main traits breeders that are focused on. Flowering time, which depends on the weather conditions with a combination of chill and heat requirements, is essential for the simultaneous cross-pollination of self-incompatible cultivars; it is also key in avoiding the loss of crops due to late frosts. Chilling is in fact, essential for floral development (Sánchez-Pérez et al. 2012).

In the annual growth cycle of *Prunus* species such as almond, three states have been described: the para-, endo- and ecodormancy states. Paradormancy is due to bud growth inhibition by other organs within the tree and is linked to apical dominance. Endodormancy, also known as deep dormancy or winter dormancy, is induced during the summer and overcome by low temperatures during the autumn and the winter. During endodormancy, meristems are inactive and remain protected within the buds. Endodormancy prevents the plant from resuming growth, even when the environmental conditions are favorable. Lastly, ecodormancy is regulated by environmental factors such as heat requirements (Campoy et al. 2011, Lang et al. 1987). Previously, it has been suggested that flowering time mainly depends on endodormancy release (Egea et al. 2003), which relies on the chilling requirements (CRs) of each cultivar (Lang et al. 1987). Only when chilling and heat requirements (HR) are fulfilled, does flower development resume and flowering take place; in *Prunus*, this occurs mainly during spring (Fadón et al. 2018), but also during winter in almond.

In temperate climates, moderate temperatures interspersed between cold periods also contribute to chill accumulation (Erez and Couvillon 1987). Although insufficient chill accumulation can allow for endodormancy release (Benmoussa et al. 2017, Prudencio et al. 2018a), it is usually accompanied by abnormal flower bud development, which has a negative effect on production (Andreini et al. 2012, Azizi Gannouni et al. 2017, Legave et al. 2013). The lack of chilling is of increasing significance under the climate change scenario predictions (Luedeling et al. 2009, Ponti et al. 2014, Rodríguez et al. 2019).

Nevertheless, studies on the genetic control of endodormancy release and flowering time are still scarce (Sánchez-Pérez et al. 2014). In almond, CRs and flowering time have been described as polygenic traits, and the variance of the first can be explained by a major Quantitative Trait Loci (QTL) called *Late blooming (Lb)* (Ballester et al. 2001, Sánchez-Pérez et al. 2012). The quantitative inheritance of CRs has also been observed in peach (Fan et al. 2010), sweet cherry (Castède et al. 2015) and apricot (Salazar et al. 2016) flowerbuds.

The first genes described linking dormancy and flowering in *Prunus* species were *DORMANCY ASSOCIATED MADS-BOX* (*DAM*) genes (Bielenberg et al. 2008, Rothkegel et al. 2017, Xu et al. 2014, Yamane et al. 2011, Zhao et al. 2018, Zhu et al. 2015). Moreover, genes related to dormancy induction and maintenance by cold in almond (*P. dulcis*), such as *P. dulcis GIGANTEA* (*PdGIGANTEA*) and *P. dulcis C-REPEATED BINDING FACTORs* (*PdCBFs*), have also been studied (Barros et al. 2012, Barros et al. 2017). More recently, a specific class III peroxidase gene has been cloned and analyzed during the endodormancy to ecodormancy transition (Prudencio et al. 2019).

Because of its economic importance, endodormancy release is being studied in model and cultivated tree species. Although the whole mechanism that regulates this process is not entirely known yet (Ding and Nilsson 2016), stimuli for dormancy release as chill accumulation or dormancy-breaking agents were found to converge in four main interconnected pathways: hormone regulation, redox status, metabolic changes and cell transport (Beauvieux et al. 2018). In the last decade, high-throughput sequencing technology has made it possible to use the transcriptomic approach to decipher which gene networks are working upon dormancy onset and release (Bai et al. 2013, Howe et al. 2015, Ionescu et al. 2017, Zhang et al. 2018). The availability of almond cultivars with a wide range of flowering times (Martinez-Gomez et al. 2017) thus offers valuable plant material for dormancy studies.

In this work, in the search for candidate genes for dormancy monitoring in almond, we compared flower bud transcriptomes from three cultivars with different CRs and flowering times (extra-early, extra-late and ultra-late), at different dormancy states (A-AB-B). Genes related to sugar synthesis and mobilization, lipid peroxidation, coumarate metabolism, transmembrane transport, cell wall remodeling and abscisic acid (ABA) synthesis and signaling, together with *DAM*-like genes, were selected for quantitative real-time PCR (qRT-PCR) analysis in two consecutive seasons, to evaluate whether they could be used either to discriminate between early- and late-flowering cultivars or as general almond dormancy release markers. This knowledge could lead to the identification of early and late flowering time candidate genes that might give clues about differential chilling responses or other cultivar-specific traits of almond species.

## Materials and methods

### Plant materials

‘Desmayo Largueta’ [extra-early flowering with a mean CR of 18 Chill Portions (CP)], ‘Penta’ (extra-late, 47 CP) and ‘Tardona’ (ultra-late, 55 CP) were the three cultivars used in this study (Table S1 available as Supplementary data at *Tree Physiology* Online). Ten-year-old trees were grafted onto Garrigues seedling rootstock and then grown in the drip-irrigated experimental orchard of CEBAS-CSIC (Santomera, Murcia, Spain). ‘Penta’ (‘S5133’ × ‘Lauranne’) and ‘Tardona’ (‘S5133’ × ‘R1000’) are releases from the CEBAS-CSIC breeding program that combine late-flowering sources from Italy, France and Ukraine: ‘Lauranne’ is ‘Ferragnés’ × ‘Tuono’; ‘S5133’ is an open pollination of ‘Primorskii’, an Ukrainian cultivar; and ‘R1000’ is ‘Tardy Nonpareil’ × ‘Tuono’. ‘Tardona’ also presents the spontaneous *Lb* mutation from the Californian cultivar ‘Tardy Nonpareil’. Finally, ‘Desmayo Largueta’ is a landrace from Spain traditionally cultivated in the Ebro Valley, but nowadays, it is cultivated throughout the country.

**Figure 1.**
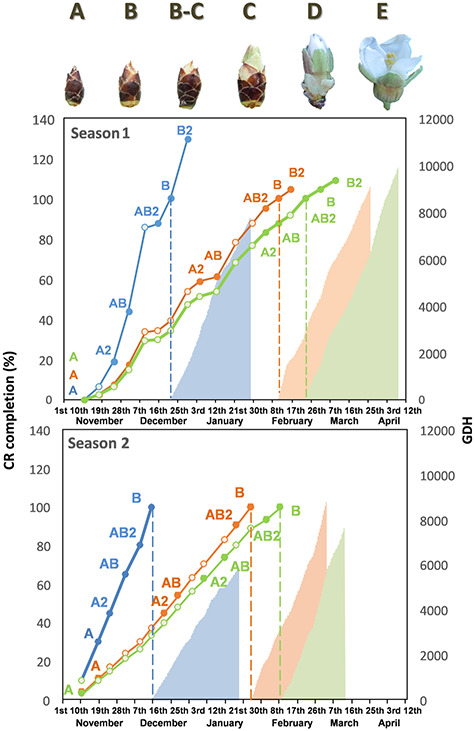
The phenological states of almond flower buds and sampling schedule of the two seasons of study of the extra-early Desmayo Largueta (blue), extra-late Penta (orange) and ultra-late Tardona (green) almond cultivars. CR% completion is indicated for each cultivar by lines and the GDHs for flowering are indicated by areas. Flower bud samples harvested in different phenological states (A, A2, AB, AB2 and B) are represented by colored points. The samples sequenced (A, AB and B states) are from Season 1.

In this work, branches and flower bud samples from endodormancy (10/17 November) to ecodormancy (after endodormancy release) were collected from the abovementioned cultivars during 2015–16 (Season 1) and 2016–17 (Season 2) (Figure 1). Flower buds (15 flower buds per sample) were collected from the same tree per cultivar and kept on ice until being descaled and frozen at −80 °C. The samples were classified according to their natural phenological state at the moment of sampling in the field. Season 1 samples were selected for RNA sequencing and classified as either endodormant flower buds, in phenological state A, in which the flower bud is small and enclosed by brown scales, or ecodormant flower buds, in phenological state B, in which the flower bud is swollen but still enclosed by its own brown scales. The A samples were taken on the first day of sampling (10 November, 2015) for all cultivars assayed. The B samples were taken on the date at which every cultivar had fulfilled its CRs, according to previously described forcing experiments (Prudencio et al. 2018a). AB samples were intermediate samples selected when each cultivar had achieved at least 40% of their CRs. For the experimental design, it was considered that endodormancy release could take place at a certain time point between the AB and B samples (Figure 1).

### Chilling requirements for endodormancy release and heat requirements for flowering time

Chill accumulation during the two winter seasons was calculated as CP using the Dynamic model (Erez and Couvillon 1987). The hourly temperature from 1 November was recorded with a HOBO® UX100–003 placed in the experimental orchard. The CRs of each cultivar were established by the CPs accumulated from 1 November to the endodormancy release date of each cultivar (Table S1 available as Supplementary data at *Tree Physiology* Online).

The endodormancy release date was estimated by the forcing method, which consists of the following: from 1 December, three branches (mixed twigs, 1-year shoots, 40-cm long and 5 mm in diameter) of each cultivar (one tree per cultivar) were picked weekly in the field and forced to flower in a growth chamber with controlled conditions (23 ± 1 °C, relative humidity 40% and 16/8 h photoperiod). Branches were placed in jars with 5% sucrose and 0.1% aluminum sulfate solution. Before immersion, a fresh cut was made at the base of the branches. The date of endodormancy release was established when, after 10 days in the growth chamber, at least 50% of the flower buds were in the B–C state (Felipe 1977) (Figure 1).

The flowering time of each cultivar was recorded in the field when 50% of the flowers were open (Table S1 available as Supplementary data at *Tree Physiology* Online). Finally, heat accumulation was estimated between dormancy release and flowering time (ecodormancy period) according to Richardson et al. (1975) as growing degree hours (GDHs), defined as the hourly temperatures minus 4.5 °C (Figure 1).

### RNA extraction and sequencing

Total RNA was extracted according to a standardized protocol (Le Provost et al. 2007). RNA samples were treated with DNaseI (AMBION, Thermo Fisher Scientific, Waltham, MA, USA) and purified with a PowerClean Pro RNA Clean-Up kit (MOBIO, Jefferson city, MO, USA). Samples containing 2–5 μg of RNA were sent to ‘Sistemas Genómicos’ (Valencia, Spain) for library preparation and RNA sequencing. All cDNA libraries were prepared according to Illumina protocols and subsequently sequenced (125 bp paired end) using the Illumina HiSeq 2000 platform. Each library was sequenced three times—with the exception of the ‘Penta’ AB sample, which was sequenced twice—which generated, in the majority, three technical replicates for statistical analysis.

### De novo transcriptome assembly

RNA sequencing (RNA-seq) generated 934 million reads, approximately 100 million reads per sample (Table S1 available as Supplementary data at *Tree Physiology* Online). After performing quality control using FastQC software and trimming the reads, a 628 million 125 bp paired-end read was used to do a *de novo* assembly. This assembly, consisting of 68,361 gene fragments (contigs), was used as a reference for gene expression analysis. The average contig length was 854 bp (Table S2 available as Supplementary data at *Tree Physiology* Online). Between 73 and 77% of all reads could be mapped to this reference. Trimming, mapping and *de novo* assembly were done using CLC Genomics Workbench 5 (QiAGEN, Hilden, Germany). All assembled transcripts were mapped against the *P. dulcis* reference transcripts (Sánchez-Pérez et al. 2019) by BLASTN, setting a minimum *E*-value of 0.0001, and the gene annotation was associated with each ‘Prudu.’ gene code (Table S2 available as Supplementary data at *Tree Physiology* Online).

To functionally categorize all *P. dulcis* contigs in the assembled reference, Gene Ontology (GO) terms were assigned to each contig using Blast2GO software (Table S3 available as Supplementary data at *Tree Physiology* Online). Gene Ontology terms provide a controlled vocabulary for describing the functions of genes across species. Blast2GO is an automated tool for the assignment of GO terms based on sequence similarity (Conesa et al. 2005).

### Differentially expressed genes identification, clustering and GOEA

**Figure 2.**
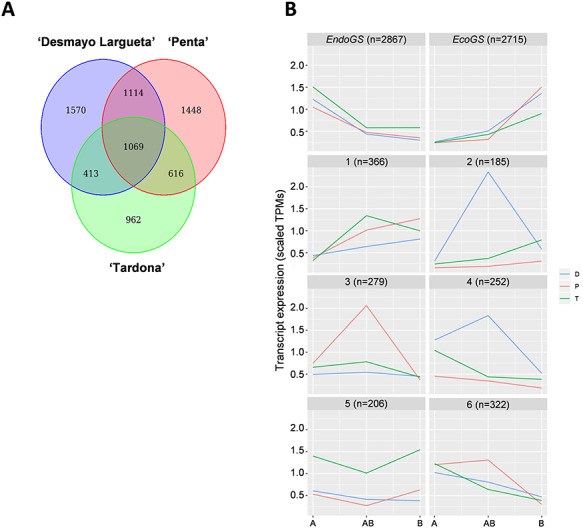
Almond DEGs identified during dormancy release (A, AB, B samples) in the three cultivars of study. (A) Common and specific DEGs among cultivars represented by a Venn diagram. (B) DEGs clusters represented by scaled TPM of genes included within each cluster. The number of genes per cluster (*n*) is indicated. Endodormant and Ecodormant Genes Superclusters are considered *EndoGS* and *EcoGS*, respectively. The extra-early flowering Desmayo Largueta cultivar (‘D’) is in blue; the extra-late flowering Penta cultivar (‘P’) is in red and the ultra-late flowering Tardona cultivar (‘T’) is in green.

The quality of raw Illumina FastQ was evaluated with FastQC (Andrews et al. 2012), and poor quality reads (minimum length of 35 bp, minimum Phred-quality score of 25) were removed using BBDuk (BBTools, https://jgi.doe.gov/data-and-tools/bbtools/). High-quality reads were pseudo-aligned against the *de novo* assembled transcriptome and counted with Kallisto (Bray et al. 2016). Statistical analysis was performed with NOISeq (Tarazona et al. 2015) using the Trimmed Mean of M-values (TMM) normalization method (Robinson and Oshlack 2010) and using the following command: noiseq (counts.noiseq, norm=`tmm', factor=`Group', pnr=0.2, nss=5, v=0.02, lc=1, replicates=`technical'). Only the genes with a probability (*q*-value) of differential expression >0.95, which would be equivalent to selecting below 0.05 by false discovery rate, and a fold change (FC) ≥|2|, were considered as statistically significant. Following this criteria, common and specific differentially expressed genes (DEGs) were identified among cultivars (Figure 2A).

Differentially expressed genes among the A, AB and B samples of the three cultivars studied and the corresponding log_2_FC and *q*-values are shown in Table S4 available as Supplementary data at *Tree Physiology* Online, along with the almond gene and GO annotation. Differentially expressed genes were grouped into similar expression profile clusters using *K*-means (Hartigan and Wong 1979) and transcripts per million (TPM) (Wagner et al. 2012) values as input, scaled for each gene across all samples (Figure 2B). The number of clusters (*K* = 8) was selected to optimize four different stability measures [average proportion of non-overlap; average distance (AD); AD between means; and figure of merit]. The clustering stability was evaluated by comparison within the same clustering but removing one column at a time. A heatmap of the DEGs within clusters was constructed using scaled TPM (Figure S1 available as Supplementary data at *Tree Physiology* Online). Transcription variation, expressed as scaled TPM of genes identified within each cluster, is shown in Table S5 available as Supplementary data at *Tree Physiology* Online, as is contig annotation in the almond genome.

Following differential expression analysis, GO Enrichment Analysis (GOEA) was performed using hypergeometric tests (Tian et al. 2017) on the eight clusters defined before.

### Identification of orthologous genes

In addition to DEG selection according to statistical criteria, the 68,361 translated contigs from the abovementioned almond assembly and 43,673 protein sequences from the sweet cherry genome *Prunus*_*avium*_v1.0 (Shirasawa et al. 2017) were compared with a collection of 250 dormancy-related translated contigs from kiwifruit (Balk et al. 2017) to identify orthologous sequences using OrthoMCL software (Li et al. 2003). The reason for using kiwifruit and sweet cherry was to obtain other genes related to dormancy in temperate fruits that could not have been selected by the criteria previously mentioned. For the IntraBlast analysis, a threshold *E*-value of 1 × 10^−5^ was used, and the OrthoMCL inflation factor used was 1.8. This resulted in 147 protein groups that contained, at least, one sequence from each species.

### qRT-PCR

For each cultivar assayed, RNA-seq data were validated by qRT-PCR using two biological replicates, representing two different seasons, Season 1 and Season 2. Sequenced samples (A, AB and B) from Season 1, together with the intermediate samples collected between them (A2, AB2 and B2), were used to validate the RNA-seq data obtained by qRT-PCR in three technical replicates. A2 and AB2 samples are previous to AB and B samples, respectively. In addition, samples from Season 2 (A, A2, AB, AB2, B and B2) were classified following the criteria explained in the, Plant materials, section and were also analyzed (Figure 1). Therefore, the qRT-PCR experiment was performed in the three cultivars object of study, in five different states, in two different seasons representing two biological replicates, with three technical replicateseach.

Candidate DEGs selected for RNA-seq validation are shown in Table 1. The primers were designed using CLC Genomics Workbench 5, preferably on exon–exon junctions to ensure the absence of genomic DNA amplification. The following adjustments were used for primer design: a PCR product size of 75–160 bp and a primer melting temperature (Tm) of 54–60 °C; the other adjustments were kept in the default mode. The primers used for qRT-PCR are listed in Table S6 available as Supplementary data at *Tree Physiology* Online. Only primers with an efficiency >80% were used for qRT-PCRs. Primer efficiency was tested by the standard curve method. The qRT-PCR was performed in 10-μl reactions of 2 × SYBR GREEN PCR mix using the StepOnePlus® PCR system of Applied Biosystems. A total of 5-ng cDNA and 2.5 μM of each set of primers were added. Relative expression was determined considering the efficiency of each primer pair (Pfaffl 2001) using *60S* and *OEP16* as housekeeping genes for data normalization.

**Table 1:** Dormancy candidate genes mapped onto the almond genome (DDBJ database: AP019297-AP019304) and whose expression was analyzed by qRT-PCR. Candidate genes are classified in the *EcoGS* (eco) or *EndoGS* (endo), or by cluster number. Genes whose orthologs are found in other species with differential expression pattern during dormancy as in almond are indicated with a ‘yes’ in the OrthoMCL column. The contig sequences and sizes can be consulted in Table S2, available as Supplementary data at *Tree Physiology* Online, with the Contig ID. Gene name used in this work and gene annotation associated are indicated. A *Prunus persica* homolog code was included according to top BLAST hit results against peach genome v2.0.a1 (*E*-value is shown)

*P. dulcis* code	Cluster	OrthoMCL	Contig ID	Gene name	Gene annotation	*P. persica* code	*E*-value
Prudu.01G134200	Eco	No	58,535	*NIP7*	AQUAPORIN, NODULIN-LIKE INTRINSIC PROTEIN 7	Prupe.1G152300.1	4.04E−93
Prudu.01G238900	Eco	Yes	49,289	*GLUCAN ENDO-β-1,3-GLUCOSIDASE*	GLUCAN ENDO-β-1,3-GLUCOSIDASE	Prupe.1G252400.1	0
Prudu.01G481600	Endo	No	563	*DAM*-like 1	DORMANCY ASSOCIATED MADS BOX 1-like 1	Prupe.1G531100.2	9.87E−79
Prudu.01G481600	Endo	No	569	*DAM*-like 2	DORMANCY ASSOCIATED MADS BOX 2-like 2	Prupe.1G531100.1	0
Prudu.02G194600	3	Yes	25,446	*ATHB12*	ARABIDOPSIS THALIANA HOMEOBOX-LEUCINE ZIPPER PROTEIN 12	Prupe.2G194400.1	0
Prudu.03G036100	Endo	No	1,494	*LOX3.1*	LINOLEATE 13S-LIPOXYGENASE 3–1, CHLOROPLASTIC	Prupe.3G039200.1	0
Prudu.03G257900	Endo	Yes	10,351	*RS5*	GALACATINOL-SUCROSE GALACTOSYLTRANSFERASE 5, RAFFINOSE SYNTHASE 5	Prupe.3G289900.1	0
Prudu.04G070600	Endo	Yes	21,024	*NCED5*	NINE-CIS-EPOXYCAROTENOID DIOXYGENASE 5, ABA BIOSYNTHESIS	Prupe.4G082000.1	0
Prudu.05G071900	Eco	No	63,835	*AIP2*	ABI3 INTERACTING PROTEIN 2, E3 LIGASE	Prupe.5G067900.1	0
Prudu.05G129900	Eco	Yes	45,643	*EG1*	ENDO-β-1,4-GLUCANASE	Prupe.5G131300.1	0
Prudu.05G142100	Eco	Yes	64,531	*SWEET10*	BIDIRECTIONAL SUGAR TRANSPORTER SWEET10	Prupe.5G146500.1	0
Prudu.08G187400	Eco	Yes	48,215	*XET*	XYLOGLUCAN ENDOTRANSGLUCOSYLASE 2	Prupe.8G200900.2	0
Prudu.08G242500	Eco	No	51,349	*4CLL1*	4-COUMARATE:CoA LIGASE-LIKE 1	Prupe.1G224800.1	0

To validate RNA-seq expression data, the Pearson correlation index (*r*) was calculated between the TPM values of sequenced samples (A, AB and B) and the qRT-PCR values of the same samples from Season 1 for each cultivar (Table S7 available as Supplementary data at *Tree Physiology* Online). In addition, to check if gene expression patterns of candidate genes are conserved from one season to another, *r* values between qRT-PCR values from Season 1 and Season 2 (A, A2, AB, AB2 and B samples) were calculated as well (Table S7 available as Supplementary data at *Tree Physiology* Online).

### Localization of candidate genes in the almond genome

Candidate genes analyzed in this study (Table 1) and the simple sequence repeat (SSR) markers BPPCT011, CPDCT027, CPPCT002, CPPCT008, CPSCT042, M15a, PceGA025, pchgms238, pchgms29, UDA-045, UDAp418, UDAp439, UDP-96003, UDP-96013, UDP-96008, UDP-97402b and UDP-98411 shown in a previous paper by Sánchez-Pérez et al. (2012) were localized using CLC Genomic Workbench 20 software, together with the QTL for CRs, HRs and flowering time, previously described by Sánchez-Pérez et al. (2012, 2014), by searching for one of the primer pair sequences corresponding to each of the SSR markers.

## Results

### Chilling requirements for endodormancy release and heat requirements for flowering time

The chilling accumulation, expressed as the percentage (%) of the CRs of each cultivar, and the HRs for flowering of the three cultivars studied in Season 1 and Season 2 are indicated in Figure 1. The CP accumulation progression over the sampling period and the flowering time of every cultivar studied in Season 1 and Season 2 are indicated in Table S1 available as Supplementary data at *Tree Physiology* Online.

### Differentially expressed genes identification and clustering

Dormancy-related DEGs were identified by pair-wise comparison over time, from endodormancy to ecodormancy (A-AB to B). After identification, the 7191 DEGs were subjected to clustering. This resulted in Endodormancy and Ecodormancy Genes Superclusters (*EndoGS* and *EcoGS*, respectively) and six additional clusters (named Clusters 1–6) (Figure 2B and Table S5 available as Supplementary data at *Tree Physiology* Online). *EndoGS* (2867 DEGs) grouped genes that were downregulated with endodormancy release (AB and B samples) accounted for almost half of the genes (47%). In contrast, genes grouped in the *EcoGS* (2715 DEGs) were upregulated along ecodormancy (B) and sometimes in the endodormancy period as well (AB) (Figure S1 available as Supplementary data at *Tree Physiology* Online). Clusters 2 and 4 showed different expression patterns between the extra-early and the late cultivars, mainly in the AB state, in which the extra-early cultivar showed upregulation, while the late cultivars were either less upregulated (Cluster 2) or downregulated (Cluster 4) (Figure 2B and Figure S1 available as Supplementary data at *Tree Physiology* Online). Cluster 3 (279 DEGs) genes presented higher expression levels in the extra-late Penta cultivar samples than in the rest of the cultivars, in the AB state. Cluster 5 (206 DEGs) exhibited a repression in expression in the AB state, and there were higher TPM for the extra-late Tardona cultivar samples. The expression levels decreased from the A to AB and B states in Cluster 6 (322 DEGs), except in the extra-late Penta cultivar, in which there was an induction of expression levels in the AB state (Figure 2B and Figure S1 available as Supplementary data at *Tree Physiology* Online).

### Gene Ontology Enrichment Analysis among clusters

**Figure 3.**
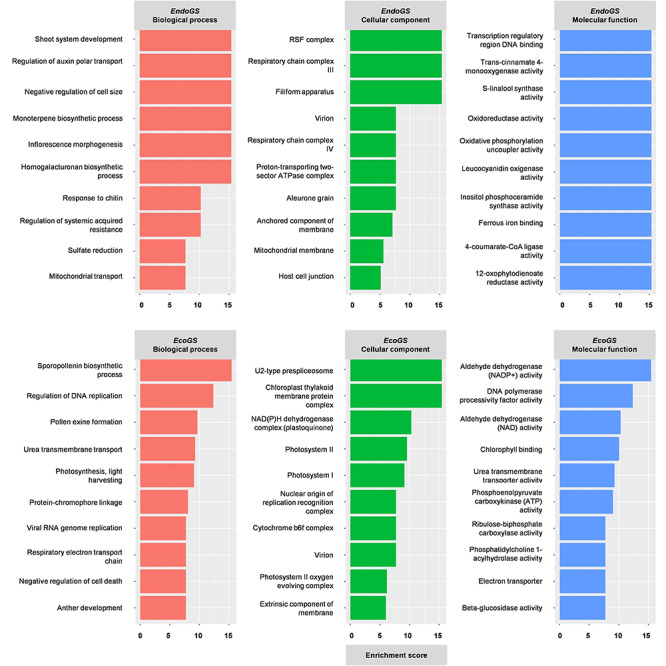
Gene Ontology categories related to the enrichment score in *EcoGS* (top) and *EndoGS* (bottom): biological processes (red bars), cellular components (green bars) and molecular functions (blue bars).

Gene Ontology Enrichment Analysis within each cluster showed highly enriched categories specific to each cluster and also common among different clusters (Figure 3 and Figure S2 available as Supplementary data at *Tree Physiology* Online). In the case of *EndoGS*, the highly enriched categories related to biological processes were ‘shoot system development’, ‘regulation of auxin polar transport’ (also found in Cluster 4), ‘negative regulator of cell size’ and ‘inflorescence morphogenesis, (both also found in Clusters 2 and 4). The most enriched category in the *EcoGS* was ‘sporopollenin biosynthetic process’ (common to Clusters 1 and 3), followed by ‘regulation of DNA replication’, ‘pollen exine formation’ and ‘anther development’, among others (Figure 3).

We found many GO categories in the *EndoGS* related to molecular function. We would like to highlight the presence of ‘S-linalool synthase activity’, which was also present in Clusters 1 and 3, and ‘inositol-phosphoceramide (IP) synthase activity’ also found in Clusters 1, 3 and 5 (Figure 3). The other remarkable categories, ‘4-coumarate-CoA ligase activity’ and ‘trans-cinnamate-4-monooxygenase activity’, were also present in Cluster 4 (Figure 3 and Figure S2 available as Supplementary data at *Tree Physiology* Online). Such enzymatic activities are involved in the phenylpropanoid synthesis pathway.

Gene Ontology categories of cellular components were generally not specific to *EndoGS* or *EcoGS* (Figure 3). Among them, in Clusters 1 to 3, we could find the *EndoGS* categories ‘RSF complex’, ‘respiratory chain complex III’ and ‘filiform apparatus’, and the *EcoGS* categories ‘U2-type presliceosome’ and ‘chloroplast thylakoid membrane protein complex’ (Figure 3 and Figure S2 available as Supplementary data at *Tree Physiology* Online).

### Candidate genes

**Figure 4.**
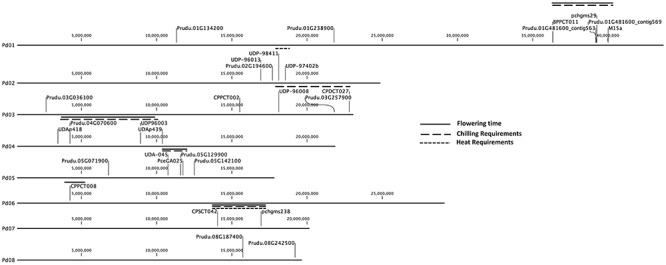
Position in the almond genome of the 13 genes identified in this study, as described in Table 1, and the SSR markers described by Sánchez-Pérez et al. (2012, 2014): BPPCT011, pchgms29 and M15a in G1; UDP-96013, UDP-98411 and UDP-97402b in G2; CPPCT002, UDP-96008 and CPCT027 in G3; UDAp-418, UDAp-439 and UDP-96003 in G4; UDA-045 and PceGA045 in G5; CPPCT008 in G6; and CPST042 and pchgms238 in G7, located in the identified QTL linked to flowering time, CRs and HRs.

Thirteen candidate genes were selected from those present in *EndoGS* or *EcoGS*, and their orthology to dormancy-related genes from kiwifruit and sweet cherry (Table 1). Those candidate genes showed a similar expression pattern to that of kiwifruit and sweet cherry in dormancy RNA-seq experiments (Balk et al. 2017). From the OrthoMCL analysis, candidate genes were selected not only by their relationship with dormancy release processes (such as lipid and coumarate metabolism, transmembrane transport and ABA signaling) but also by the relative position of the candidate genes shown in Figure 4 (this is the case of the *DAM*-like genes), as explained below.

The positions of the endodormancy and ecodormancy candidate genes described in Table 1, and validated by qRT-PCR, were localized in the almond genome, together with the SSR markers and linked to dormancy QTL (chilling and heat requirements and flowering time) described previously in Sánchez-Pérez et al. (2012, 2014) (Figure 4).

#### Endodormancy-associated genes

**Figure 5.**
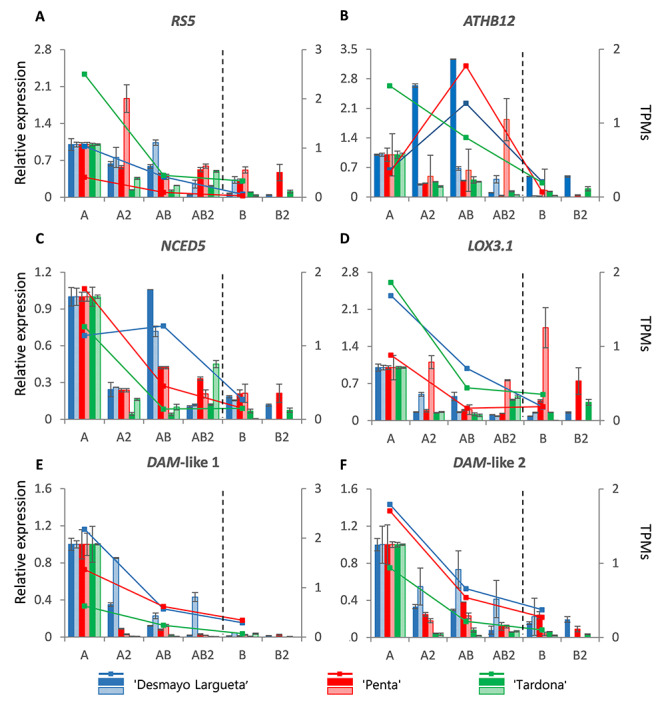
Endodormancy genes (A–F). Expression dynamics of candidate DEGs whose expression was higher during the endodormancy period (``Desmayo Largueta'': blue; ``Penta'': red; ``Tardona'': green). Endodormancy release (between the AB2 and B state) is indicated by a dashed line. The relative expression of genes by qRT-PCR (bars) is represented by means of three technical replicates of RNA pools from Season 1 (colored bars) and Season 2 (bars with colored lines). The TPM values (colored lines) were obtained from RNA-sequenced samples (A, AB, B), by means of three sequencing replicates of RNA pools from Season 1.

The endodormancy-asso-ciated genes were selected as displaying higher expression during endodormancy (A to AB samples) and lower expression during ecodormancy (AB to B samples) during Season 1 and Season 2 (Figure 5). Such candidate genes were mainly classified within the *EndoGS* (Table 1 and Figure 2B). Among these genes, we found a *RAFFINOSE SYNTHASE 5* (*RS5*) ortholog involved in carbohydrate metabolism (Figure 5A). In some cases, during Season 2, downregulation with respect to the A state is not observed until the AB (Penta cultivar) or the AB2 (Desmayo Largueta cultivar) state. In case of Penta cultivar, this reduced the *r* value between qRT-PCR results of the two seasons (Table S7 available as Supplementary data at *Tree Physiology* Online). However, there was high correlation between qRT-PCR and RNA-seq results in all the cultivars studied (Figure 5A).

Several ABA-related genes were also analyzed, most of which were selected for their orthology with dormancy-release relevant genes in other species, as evidenced by OrthoMCL analysis (see Table 1). These genes included: *Arabidopsis thaliana HOMEOBOX-LEUCINE ZIPPER PROTEIN 12* (*ATHB12*) and *NINE CIS EPOXYCAROTENOID DIOXYGENASE 5* (*NCED5*) almond orthologs (Figure 5B and C, respectively). *ATHB12* is the only transcript included in this selection of endodormancy-associated genes not present in the *EndoGS*, but in Cluster 6 (Table 1). This is because the *ATHB12* expression pattern depends on the cultivar and season, as reflected by the different correlation values obtained (Table S7 available as Supplementary data at *Tree Physiology* Online). According to RNA-seq (Season 1), the extra-early Desmayo Largueta and extra-late Penta cultivars showed increased expression levels before endodormancy release and decreased levels afterwards, whereas the ultra-late Tardona cultivar showed a continuous downregulation. RNA-seq and qRT-PCR data highly correlate in *NCED5*, as well as qRT-PCR assays from different seasons (Table S7 available as Supplementary data at *Tree Physiology* Online). In Desmayo Largueta cultivar, expression levels increased in the AB state and were even greater than A state levels in Season 1 (Figure 5C).

Another candidate gene included in this group was *LINOLEATE 13S-LIPOXYGENASE 3–1* (*LOX3.1*) (Figure 5D). According to RNA-seq, *LOX3.1* expression decreased from the A to the AB state, which was corroborated by qRT-PCR in all cases. Expression levels remained constant from AB to B and even increased according to qRT-PCR results, especially in the extra-late Penta cultivar during Season 2, (Figure 5D), which decreased the *r* value with Season 1 results (Table S7 available as Supplementary data at *Tree Physiology* Online).

Finally, *DAM*-like expression levels were continuously downregulated during endodormancy release in all cultivars (Figure 5E and F). The downregulation was greater in late-flowering cultivars (Penta and Tardona) than in the early-flowering cultivar (Desmayo Largueta). TPM highly correlated with qRT-PCR values (*r* ≤ 0.96), and we also obtained high correlations (*r* ≤ 0.84) between qRT-PCR values from both seasons of study (Table S7 available as Supplementary data at *Tree Physiology* Online).

#### Ecodormancy-associated genes

**Figure 6.**
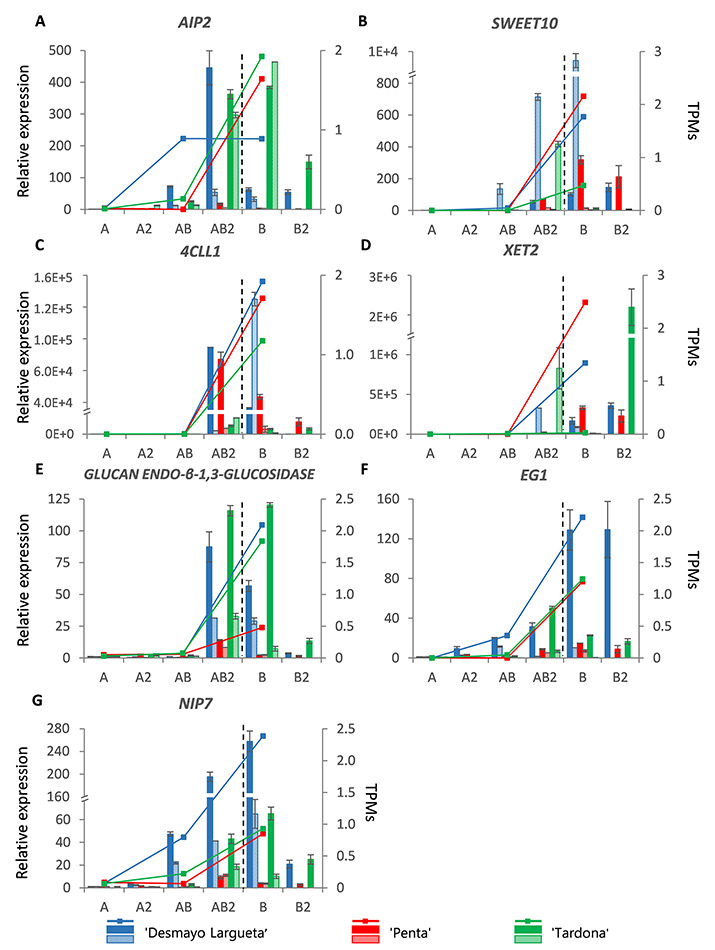
Ecodormancy genes (A–G). Expression dynamics of candidate DEGs whose expression was higher during the ecodormancy period (``Desmayo Largueta'': blue; ``Penta'': red; ``Tardona'': green). Endodormancy release (between the AB2 and B state) is indicated by a dashed line. The relative expression of genes by qRT-PCR (bars) is represented by means of three technical replicates of RNA pools from Season 1 (colored bars) and Season 2 (bars with colored lines). The TPM values (lines) were obtained from RNA-sequenced samples (A, AB, B), by means of three sequencing replicates of RNA pools from Season 1.

*Ecodormancy associated* genes included in the *EcoGS* (Figure 2B) displayed the highest expression during ecodormancy (AB to B samples) and a lower expression during endodormancy (A to AB samples) in both seasons studied (Figure 6). In all these genes, qRT-PCR values and RNA-seq highly correlated (*r* ≤ 0.84) (Table S7 available as Supplementary data at *Tree Physiology* Online). This is the case of the *ABI3 INTERACTING PROTEIN 2* (*AIP2*) almond ortholog (Figure 6A), whose maximum expression levels were observed in the endodormancy (AB2) to ecodormancy (B) transition.

A similar expression pattern was observed for *SWEET10* and *4-COUMARATE:CoA LIGASE-LIKE 1* (*4CLL1*) almond orthologs (Figure 6B and C). An expression level induction was observed just before endodormancy release. The moment in which maximum expression levels were reached varied from AB2 to B state from Season 1 to Season 2 in ``Desmayo Largueta'', which decreased the *r* value between qRT-PCR results of the two seasons of study (Table S7 available as Supplementary data at *Tree Physiology* Online).

Other genes included in *EcoGS* coded for cell wall remodeling enzymes such as *XYLOGLUCAN ENDOTRANSGLUCOSYLASE 2* (*XET2*), *GLUCAN ENDO-β-1,3-GLUCOSIDASE* and *ENDO-β-1,4-GLUCANASE* (*EG1*) (Table 1). The relative expression levels of *XET2* increased from AB2 to B in the extra-early and the extra-late cultivars, and there was a delay in the ultra-late Tardona cultivar in Season 1 (Figure 6D). The moment in which maximum expression levels were reached varied from AB2 to B2 state from Season 1 to Season 2 in Tardona cultivar, which turn negative the *r* value between qRT-PCR results (Table S7 available as Supplementary data at *Tree Physiology* Online). *GLUCAN ENDO-β-1,3-GLUCOSIDASE* expression levels peaked in the AB2/B states in all cultivars in both seasons (Figure 6E). Finally, *EG1* showed a progressive increase from the A state; this increase was continuous in the extra-early Desmayo Largueta cultivar and up until AB2 in the ultra-late Tardona cultivar (Figure 6F).

The last ecodormancy-associated gene is *NODULIN-LIKE INTRINSIC PROTEIN 7* (*NIP7*), the almond ortholog of a gene involved in transmembrane transport (Figure 6G). In this case, expression kept increasing until the B state (or AB2 in the extra-late Penta cultivar).

The candidate genes described above grouped in the *EcoGS*, characterized by the increase of expression levels of transcripts from the AB to B state samples (Figure 2B). However, some of the genes presented contrasting expression levels in a cultivar-dependent manner, according to TPM and qRT-PCR results. This is the case of genes, such as *SWEET10*, *4CLL1* and *XET2*, which showed lower expression levels in the ultra-late Tardona cultivar samples than in the others (Figure 6B–D), and in the case of *GLUCAN ENDO-β-1,3-GLUCOSIDASE*, which showed relatively low expression levels in the extra-late Penta cultivar samples (Figure 6E). Finally, the following two genes presented higher expression levels in the early flowering Desmayo Largueta cultivar than in the extra-late Penta and ultra-late Tardona cultivars: *EG1* and *NIP7* (Figure 6F and G).

## Discussion

Transcriptional reprogramming is one of the mechanisms that leads to developmental transitions in plants (Kaufmann et al. 2010). In this case, RNA-seq technology allowed us to study transcriptomic changes in flower buds developing from the endodormant to ecodormant state (Figure 1). Following bioinformatic analysis, we obtained DEGs classified in different clusters, which showed the comparison between cultivars with different CRs and flowering times (Figure 2). As mentioned in the results section, expression differences among cultivars were mainly observed in the AB state (Figure S1 available as Supplementary data at *Tree Physiology* Online), when at least 40% of the CRs of each cultivar had accumulated but phenological evolution assessed by the forcing method did not account for dormancy release. Finally, based on the clusters-derived GOEA and the expression analysis of the selected candidate DEGs, we can therefore discuss the results obtained regarding endodormancy release in the three cultivars assayed.

### Gene Ontology Enrichment Analysis of clusters

#### Gene Ontology categories enriched in *EndoGS*

Regarding the GO categories enriched in *EndoGS*, we must mention among Molecular Functions ‘S-linalool synthesis’, which was present in Clusters 1, 3 and 4. Linalool is a monoterpene compound present in aromatic herbs, flowers and fruits of many species that contributes to scent and flavor properties (Knudsen et al. 2006, Lewinsohn et al. 2001). The GO category ‘monoterpene biosynthesis’ is among the most enriched Biological Processes in *EndoGS*. All this evidence supported an important role for linalool synthesis mainly in endodormancy.

On the other hand, IP is a known precursor of sphingolipids. The ‘IP-synthase activity’ is one of the most enriched GO categories in *EndoGS* and Clusters 1, 3 and 5. In Arabidopsis, the sphingolipidome composition of pollen is different from that of leaf samples, showing extensive glycosylation of IP-derivatives (Luttgeharm et al. 2015). Sphingolipids can act as signaling molecules in processes like Programmed Cell Death (PCD) (Liang et al. 2003). We must also mention the enrichment of negative regulation of cell death in the *EcoGS* Biological Processes. Moreover, Teng et al. proposed that sphingolipids regulate proper microspore development through the control of PCD, based on results obtained working with Arabidopsis mutants (Teng et al. 2008). Programmed Cell Death has been associated with dormancy release in the flower buds of the extra-early Desmayo Largueta and the extra-late Penta cultivars, by the differential gene methylation of apoptotic ATPases in A versus B state samples (Prudencio et al. 2018b). Indeed, as reflected by genes differentially expressed in almond flower buds, IP is an important molecule in the endodormancy and ecodormancy processes of the cultivars studied.

Another enriched GO category in *EndoGS* and Cluster 4 is ‘4-CL enzymatic activity’. Interestingly, *4CLL1* is a candidate gene analyzed in this work, showing high induction from the A to B state flower bud samples (see Candidate genes section). Phenylpropanoid compounds are thus involved in the dormancy release process, as reported by Conrad et al. (2019).

#### Gene Ontology categories enriched in *EcoGS*

We must highlight the processes linked to photosynthesis and microsporogenesis during ecodormancy, reflecting the metabolic reactivation of the bud tissue and final flower differentiation. Microsporogenesis events have been previously related to endodormancy release in *Prunus* species (Julian et al. 2011, Rios et al. 2013). One of these events is represented in GOEA as ‘pollen exine formation’, which requires the synthesis of strictosidine (Dobritsa et al. 2009). A high induction of *STRICTOSIDINE SYNTHASE-LIKE* gene was observed in our samples from endodormant to ecodormant flower buds in all the cultivars and seasons studied (data not shown), which supported functional enrichment in the *EcoGS.*

### Candidate genes

Along with gene localization, expression levels detected between RNA-seq and qRT-PCR are well correlated (*r* ≤ 0.96) in all genes, with the exception of *ATHB12*, indicating the soundness of the transcriptome sequencing.

#### Genome localization of candidate genes

Firstly, we showed the position of the markers localized in the almond genetic linkage map of ‘R1000’ × ‘Desmayo Largueta’ F1 (Sánchez-Pérez et al. 2012, 2014) and the genes found in this study. As can be seen in Figure 4, we have found genes in the chromosomes represented by pseudomolecules 1, 2, 3, 4, 5 and 8. In the QTL analyses, QTL were found in linkage groups (LGs) 1, 3, 4, 5 and 7 for CRs; in LGs 1, 4, 5 and 7 for flowering time and in LGs 2 and 7 for HRs. In LG1, where the QTL for flowering time and CR is found (between the markers BPPCT011 and M15a), *DAM*-like genes 1 and 2 were located. In LG3, between UDP96008 and CPDCT027, a QTL for CR was found and so was the gene *RS5*. In LG4, the most important QTL for flowering time and CR was found very close to UDP96003, between UDAp-418 and UDAp439, and in that position, *NCED5* was the closest gene found. In LG7, a QTL for CRs and HRs was found close to EPDCU3392, and no gene characterized here was found in G7. Despite the apparently close distance found among markers and some genes characterized in this study, fine mapping should be done. This is especially necessary and interesting in LG4 and LG1, where the main QTL for flowering time and CR are found.

#### Endodormancy genes

During endodormancy, it is considered that raffinose and other sugars, such as stachyose and galactinol, are synthesized to protect trees from chilling, drought and oxidative stress (Ibáñez et al. 2013, Nishizawa et al. 2008). In chestnut, *RAFFINOSE SYNTHASE* (*RS*) expression increased under low temperatures, and raffinose accumulated in vegetative tissues under drought stress (Nishizawa et al. 2008), which is thus in line with the downregulation of *RS5* during endodormancy release in almond (Figure 5A).

Endodormancy maintenance is mediated by different metabolites, such as ABA, which plays a role in dormancy maintenance (Li et al. 2018, Wang et al. 2015, Zhao et al. 2018). Thus, ABA-induced genes are expected to be downregulated during endodormancy release, such as *ATHB12*, a transcription factor induced by drought conditions and ABA signaling in Arabidopsis (Valdés et al. 2012). In almond, *ATHB12* ortholog gene expression was downregulated during endodormancy release, with two exceptions: in the extra-early Desmayo Largueta cultivar, this gene was induced in the AB state in Season 1, and in the extra-late Penta cultivar, it was induced in the AB2 state in Season 2 (Figure 5B). These observations point to a temporary repression of the *ATHB12* almond ortholog toward endodormancy release, probably due to ABA signaling.

On the other hand, during dormancy release, fatty acids are delivered by lipid peroxidation (Gabay et al. 2019), supplying energy and a carbon skeleton. This is important for dormancy release, since during dormancy, carbon starvation is produced and glucose is needed, not only for starch accumulation or sucrose mobilization but also for reactive oxygen species production during endodormancy release (Beauvieux et al. 2018). *LOX3.1* (Cluster 1) encodes a plastidial peroxygenase able to catalyze membrane galactolipid peroxidation and free fatty acid chains such as linolenic and linoleic acid (Pilati et al. 2014). A decrease in the expression of *LOX3.1* was observed during potato meristem tuberization (Viola et al. 2007), which is considered an analog process to dormancy release (Rodríguez-Falcón et al. 2006). Moreover, a *LOX2* transcript was downregulated under the cold treatment of Japanese apricot vegetative buds (Habu et al. 2014). Such evidence supports the decreasing pattern in the *LOX3.1* almond ortholog during dormancy release (Figure 5D), with the exception of the induction observed after dormancy release (B state) in the extra-late Penta cultivar during Season 2.

Finally, dormancy induction and maintenance has been associated with *DAM* gene family transcription in different *Prunus* species (Falavigna et al. 2019, Hao et al. 2015, Li et al. 2009, Yu et al. 2020, Zhang et al. 2018, Zhao et al. 2018, Zhu et al. 2015). Moreover, genome analysis in peach revealed six highly conserved *DAM* genes identifying a relation between the lack of expression of these genes and the absence of dormancy in evergrowing (EVG) peaches (Bielenberg et al. 2008). Such evidence is in line with the downregulation of *DAM1* and *DAM2* almond orthologs along endodormancy release observed in our study (Figure 5E and F).

#### Ecodormancy genes

As previously discussed, genes related to ABA biosynthesis and transduction showed a decreasing pattern during endodormancy release. Therefore, in the case of genes involved in the inhibition of the ABA response, transcription levels are expected to increase. This is the case of the *AIP2* almond ortholog. AIP2 has been described as a RING-type E3 ligase that targets ABI3 to 26S proteasomes for proteolysis regulation, which leads to the inhibition of the ABA response (Zhang et al. 2005). A role for this protein has never been described in bud dormancy, but a role has been described in seed dormancy in Arabidopsis (Liu et al. 2010). In wheat, in H_2_O_2_-treated seeds, a transient increase in the expression of *AIP2* was observed (Gao et al. 2012). In this work, the expression of the *AIP2* almond ortholog was found to increase during ecodormancy in RNA-seq and qRT-PCR (Figure 6A).

The enzyme encoded by *4CLL1* may be involved in the phenylpropanoid biosynthetic pathway. During cold accumulation, phenylpropanoid compounds, such as coumaric acid, accumulate during cold treatment in peach seeds (Leida et al. 2012). Once the chilling accumulation is over, coumarate could be transiently targeted for CoA ligation and downstream reactions, a reaction catalyzed by the 4-CL (4-COUMARATE:CoA LIGASE) enzyme. In this work, the *4-CL* ortholog almond transcript, *4CLL1*, was highly induced from the AB to B state (Figure 6B), which could point to a temporary activity of the encoded enzyme during dormancy release. Recently, the association of the phenylpropanoid pathway with dormancy and adaptive trait variation has been described in apricot (Conrad et al. 2019). Even more recently, phenylpropanoid compounds as cinnamic, p-coumaric, caffeic and ferulic acid have been described in the release of endodormancy in almond flower buds (Guillamón et al. 2020).

During dormancy release, regarding cell-to-cell communication, carbohydrates act not only as a source of carbon and energy, but they also function as developmental signals (Anderson et al. 2005, González-Rossia et al. 2008). For instance, sucrose accumulation and mobilization occurred in grapevine buds after dormancy release was forced by hydrogen cyanamide (HC) treatment (Ben Mohamed et al. 2012). In rose, carbohydrate signaling is required for natural endodormancy release (Rabot et al. 2012). Sucrose transporters are upregulated in such conditions, as reported in walnut (Decourteix et al. 2008), leafy spurge (Chao and Serpe 2010) and Japanese apricot (Zhang et al. 2018). Moreover, in Arabidopsis, *SWEET10* is transcriptionally activated by the flowering regulators *FLOWERING LOCUS T* (*FT*) and *SUPPRESSOR OF CONSTANS OVEREXPRESSION 1* (*SOC1*), specifically in the leaf veins (Andrés et al. 2020). All this evidence supports the dormancy release induction of the *SWEET10* ortholog in almond. However, this increase peaked rather late, at the B or B2 state (when dormancy was released or immediately afterwards), which indicates the sugar utilization capacity of buds as metabolic sinks (Beauvieux et al. 2018) (Figure 6C).

Microsporogenesis, associated with the ecodormancy phase, includes pollen exine formation, as seen in the enrichment analysis (see GOEA sections). This process is mediated by aquaporins. NIP7 belongs to a subfamily of the aquaporin proteins predominantly expressed in developing flowers in Arabidopsis*.* NIP7 has been proposed as a boric acid channel involved in pollen cell wall building in tapetal cells (Routray et al. 2018). The narrow developmental expression pattern of *NIP7* observed by Routray et al. (2018) was similar to what we observed in the almond ortholog from the AB/AB2 state to the B2 state (Figure 6D). This further supports the role of pollen development genes as one of the expression markers of the endodormancy to ecodormancy transition and therefore as potential marker candidates for almond breeding programs.

Finally, Glycosyl Hydrolase (GH)-encoding genes are well represented in this analysis. 1,3-β-Glucosidases (such as GLUCAN ENDO-β-1,3-GLUCOSIDASE), also referred to as GH17, are promoted by gibberellins (GAs) and mostly by chilling; they accumulate at plasmodesmal ends, resulting in the hydrolysis of callose sphincters (Gai et al. 2013, Rinne et al. 2011). 1,4-β-Glucanases such as EG1 (GH9) and XET2 (GH16) have an active role in the well-described cell wall remodeling and loosening (Baumann et al. 2007, Fry et al. 1992, Huang et al. 2019). The upregulation of GH-encoding genes is therefore in line with an active role of GH during dormancy release (Figure 6E–G).

Some of the ecodormancy genes described above could be used to distinguish cultivars as they presented contrasting expression levels among early and late flowering cultivars. This is the case of genes such as *NIP7* and *EG1*, whose expression might be associated with the endodormancy release date and flowering time phenotypes in almond.

## Conclusions

In this work, the natural progression from endodormancy to ecodormancy of a traditional early flowering almond cultivar (Desmayo Largueta) versus two extra/ultra-late flowering almond cultivars (Penta and Tardona) from our Almond Breeding Program was monitored by gene expression in flower buds, identifying potential dormancy-associated candidate genes related to sugar synthesis and mobilization, lipid peroxidation, coumarate metabolism, transmembrane transport, cell wall remodeling and ABA synthesis and signaling. In the endodormancy to ecodormancy transition, we highlighted the role of GH-encoding genes and *NIP7* and *SWEET10* almond orthologs, whose expression was induced before endodormancy release in all cultivars studied. As a future perspective, the DEGs detected among cultivars could be the subject of a new study to differentiate the transcriptional responses associated with CRs and flowering time. This is of great interest for agriculture, since fruit production is determined by successful flowering, which depends on climatic conditions and is severely affected by a climate-change scenario, especially in temperate regions.

## Data archiving statement

RNA-seq reads obtained have been deposited in the SRA database at NCBI (https://www.ncbi.nlm.nih.gov/sra) under the BioProject accession number PRJNA610711.

## Supplementary Material

Table_S4R1_tpaa151

Supplemental_R3_tpaa151

Table_S3R1xlsx_tpaa151

Table_S1_tpaa151

Table_S1_tpaa151
