# Supplementary material for: Identification of early and late flowering time candidate genes in endodormant and ecodormant almond flower buds
Source: Tree Physiol. 2020 Nov 16;41(4):589–605. doi: 10.1093/treephys/tpaa151 (PMC8033246; doi:10.1093/treephys/tpaa151)
Supplement: Supplemental_R3_tpaa151 [file supplemental_r3_tpaa151.pdf]

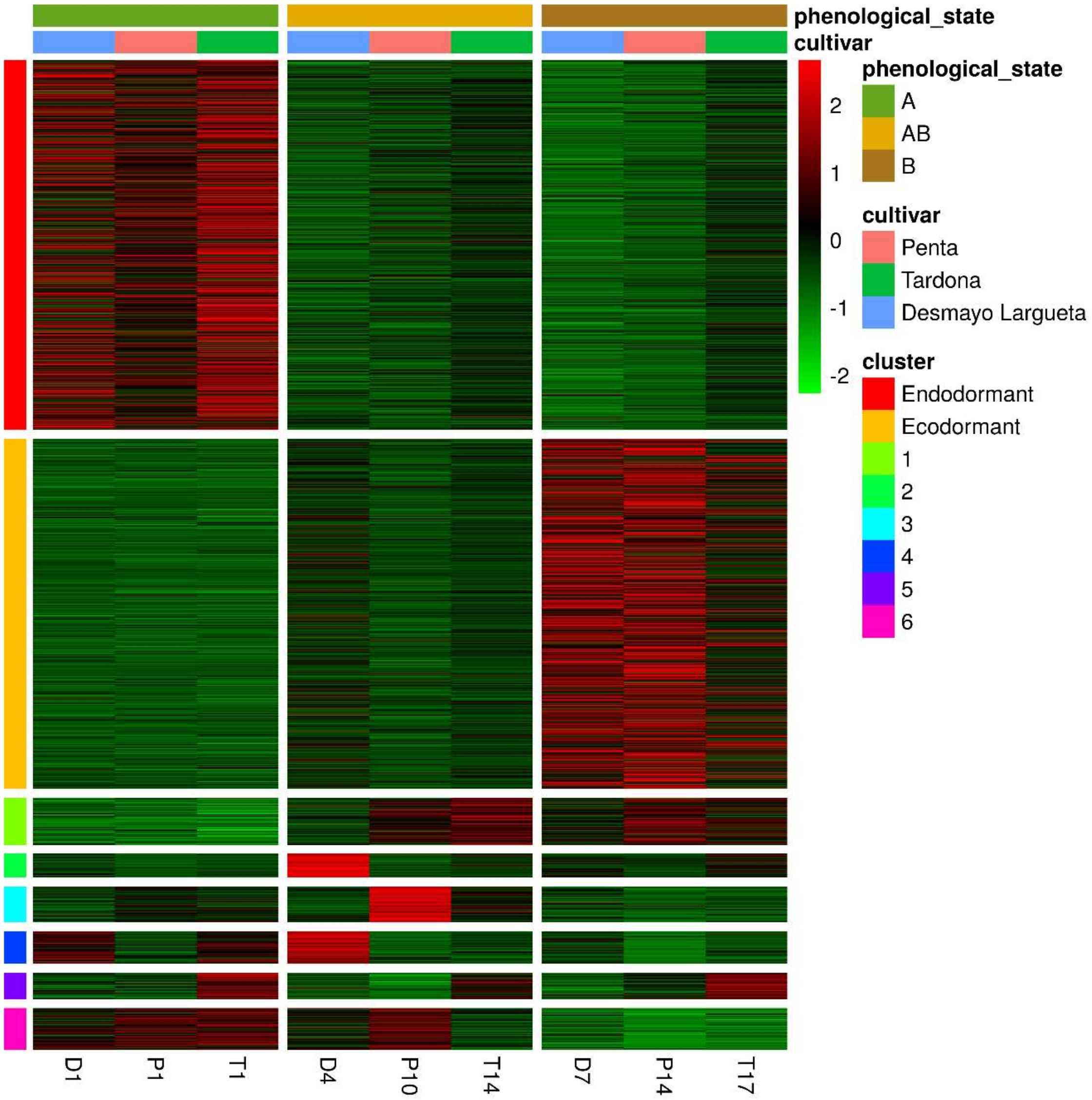

Figure S1. Heatmap of almond flower buds DEGs based on scaled TPM. Phenological states and cultivars of almond flower buds are represented by colors. A, AB and B corresponded to extra-early Desmayo Largueta (D1, D4, D7), extra-late Penta (P1, P10, P14), and ultra-late Tardona (T1, T14, T17) cultivars samples. Each cell is colored from green to red based on the level of expression of that gene in a given sample. Genes (rows) are ordered according to their cluster (*K*-means clusters 1 to 8). Clusters are ordered from *EndoGS* and *EcoGS* (top) to 1-6 (bottom) and are represented as a color bar on the left side.

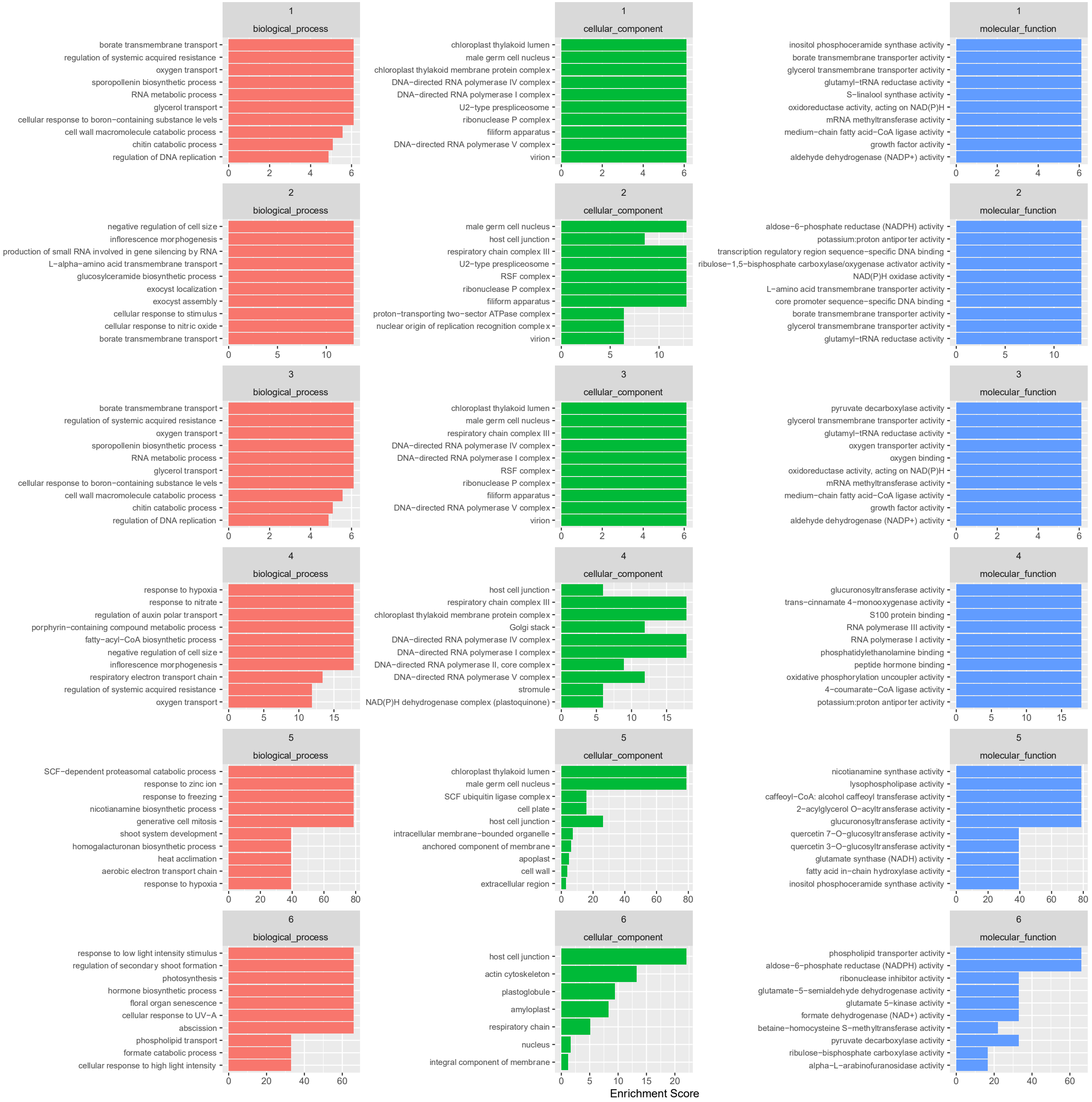

Figure S2. GO categories representation related to the enrichment score in clusters 1-6: Biological processes (red bars), cellular components (green bars) and molecular functions (blue bars).

Table S1. Experimental design for RNA sequencing and validation of almond flower buds: Samples (15 flower buds/tree/cultivar) from three cultivars with contrasting flowering time and chilling requirement (highlighted) were selected for RNA extraction and sequencing (A, AB, B) collected on Season 1 dates indicated. Samples collected during Season 2 were used together with those from Season 1 for RNA-seq validation by qRT-PCR. Million reads and reads conted (%) per sequenced sample. CP: Chill Portions.

	Flowering time (date)		Flower bud samples		Chill accumulated (CP)		RNAseq output data (Season 1)	
**Cultivar**	**Season 1**	**Season 2**	**State**	**Sampling date (Season 1)**	**Season 1**	**Season 2**	**Million reads**	**% reads counted**
Desmayo	January 28th	January 27th	A	11/10/2015	0	5.0	106.2	73.6
Desmayo			A-B	12/1/2015	7.1	9.1	121.3	73.4
Desmayo			B	12/21/2015	16.1	20.1	108.3	74.5
Penta	February 10th	February 2nd	A	11/10/2015	0	5.0	91.9	76.8
Penta			A-B	1/12/2016	25	29.2	115	74.3
Penta			B	2/10/2016	41.2	53.8	96.9	74.6
Tardona	February 23rd	February 10th	A	11/10/2015	0	5.0	90	75.4
Tardona			A-B	2/10/2016	41.2	44.8	105.3	74.4
Tardona			B	3/1/2016	49.2	60.6	99.5	75.3

Table S6. qRT-PCR primers sequence, melting temperature and amplicon size of DEGs used for RNA-seq validation. Tm: Melting temperature.

DEG name	Forward primer (5´-3´)	Tm (°C)	Reverse primer (5´-3´)	Tm (°C)	Amplicon size (bp)
XET	GGTTTGGTTCTGGGTTCT	56.16	GCTTTGTCCTTTACTCGGT	56.8	115
4CLL1	GTTCAGCAGGAGCCACTT	59.1	ACAAGAATGGATGGCTTCA	56.00	123
NIP7	TTGAAGTTTGAGGAGATGGAACAG	59.73	GGCCAATAAAGACTCGGTTCAA	60.02	111
GLUCAN ENDO-β-1,3-GLUCOSIDASE	AACTCCCTCCCTCTCCATC	59.09	CGGTGAGGTGGTTCTGGA	60.46	94
EG1	CTCCAGGGGTATATTGGG	54.82	AAGAGACCTGGAACAAAAG	53.99	148
SWEET10	GTGATGAGAAAAGTGGCAT	54.88	CCAGTGCCAACATTTTATAC	53.95	152
AIP2	ATGCAAGCGTCGTGGTAA	59.25	TGGGGTTTGTTGTTCCGTTT	59.23	93
DAM-like 1	CCTCCACTTCTTAACCAGA	55.02	TGCTCCCAGTCTCTCTCT	58.00	93
DAM-like 2	AGGAACTTGAGGAGAAGAG	54.72	TCAGTTCTTCCTCAGTTTCA	55.31	141
LOX3.1	TACGTAGCCAATGGTGTA	54.33	CGAGATCAAAGCGTGTTT	55.26	126
RS5	CTAGAAGGGGGAGAACGA	55.28	CCTAGAGAACGAGACCCA	54.94	80
ATHB12	CTGCAACTGAATAACCAAGG	56.01	TACAGCATACTCAGAGCC	54.86	90
NCED5	CTTTGGGGTGTGTTTTTCT	55.64	CTCTTCCTCCTCTTCTTCT	56.00	80
OEP16	CCACGGACCCTTTCTAAA	55.63	TGAGCACACTTTGAAGAAG	54.77	99
60S	ATACCAGTTGAAGGATCGT	55.45	GGAATATGAGTTGCTAAGAAGG	55.44	105

Table S7. Pearson correlation (*r*) values of gene expression data calculated between RNA-seq TPMs and qRT-PCR data from sequenced samples (A, AB and B) of season 1 (highlighted in grey) and the *r* values calculated between qRT-PCR data from season 1 and season 2 (A, A2, AB, AB2, B).

	**Season 1 samples (RNAseq vs qRT-PCR)**			**Season 1 vs Season 2 samples (qRT-PCR)**		
	**Desmayo**	**Penta**	**Tardona**	**Desmayo**	**Penta**	**Tardona**
RS5	0.98	0.99	1.00	0.91	0.35	0.93
ATHB12	1.00	0.00	0.98	0.20	0.01	1.00
NCED5	1.00	1.00	1.00	0.95	0.99	0.93
LOX3.1	1.00	0.98	1.00	0.83	0.22	0.98
DAM-like 1	1.00	0.98	0.96	0.84	1.00	1.00
DAM-like 2	1.00	1.00	0.99	0.87	0.98	1.00
AIP2	0.96	0.99	1.00	0.89	0.91	0.97
SWEET10	1.00	1.00	0.88	0.90	0.70	0.94
4CLL1	1.00	1.00	1.00	0.18	0.95	0.84
XET2	1.00	1.00	0.97	1.00	1.00	(-0.24)
GLUCAN ENDO-β-1,3-GLUCOSIDASE	1.00	0.96	1.00	0.98	0.97	0.73
EG1	1.00	1.00	1.00	0.58	0.97	0.89
NIP7	0.99	0.99	0.99	0.98	0.98	0.76
