# Supplementary material for: Identification of early and late flowering time candidate genes in endodormant and ecodormant almond flower buds
Source: Tree Physiol. 2020 Nov 16;41(4):589–605. doi: 10.1093/treephys/tpaa151 (PMC8033246; doi:10.1093/treephys/tpaa151)
Supplement: Table_S1_tpaa151 [file table_s1_tpaa151.docx]

|  | **Flowering time (date)** | | **Flower bud samples** | | **Chill accumulated (CP)** | | **RNAseq output data**  **(Season 1)** | |
| --- | --- | --- | --- | --- | --- | --- | --- | --- |
| **Cultivar** | **Season 1** | **Season 2** | **State** | **Sampling date**  **(Season 1)** | **Season 1** | **Season 2** | **Million reads** | **% reads counted** |
| Desmayo Largueta | January 28th | January 27th | A | 11/10/2015 | 0 | 5.0 | 106.2 | 73.6 |
| Desmayo Largueta |  |  | A-B | 12/1/2015 | 7.1 | 9.1 | 121.3 | 73.4 |
| Desmayo Largueta |  |  | B | 12/21/2015 | 16.1 | 20.1 | 108.3 | 74.5 |
| Penta | February 10th | February 2nd | A | 11/10/2015 | 0 | 5.0 | 91.9 | 76.8 |
| Penta |  |  | A-B | 1/12/2016 | 25 | 29.2 | 115 | 74.3 |
| Penta |  |  | B | 2/10/2016 | 41.2 | 53.8 | 96.9 | 74.6 |
| Tardona | February 23rd | February 10th | A | 11/10/2015 | 0 | 5.0 | 90 | 75.4 |
| Tardona |  |  | A-B | 2/10/2016 | 41.2 | 44.8 | 105.3 | 74.4 |
| Tardona |  |  | B | 3/1/2016 | 49.2 | 60.6 | 99.5 | 75.3 |

**Table S1.** Experimental design for RNA sequencing and validation of almond flower buds: Samples (15 flower buds/tree/cultivar) from three cultivars with contrasting flowering time and chilling requirement (highlighted) were selected for RNA extraction and sequencing (A, AB, B) collected on Season 1 dates indicated. Samples collected during Season 2 were used together with those from Season 1 for RNA-seq validation by qRT-PCR. Million reads and reads counted (%) per sequenced sample. CP: Chill Portions.
