# Supplementary material for: Identification of early and late flowering time candidate genes in endodormant and ecodormant almond flower buds
Source: Tree Physiol. 2020 Nov 16;41(4):589–605. doi: 10.1093/treephys/tpaa151 (PMC8033246; doi:10.1093/treephys/tpaa151)
Supplement: Table_S1_tpaa151 [file table_s1_tpaa151.pdf]

Table S1. Experimental design for RNA sequencing and validation of almond flower buds: Samples (15 flower buds/tree/cultivar) from three cultivars with contrasting flowering time and chilling requirement (highlighted) were selected for RNA extraction and sequencing (A, AB, B) collected on Season 1 dates indicated. Samples collected during Season 2 were used together with those from Season 1 for RNA-seq validation by qRT-PCR. Million reads and reads counted (%) per sequenced sample. CP: Chill Portions.

	Flowering time (date)		Flower bud samples		Chill accumulated (CP)		RNAseq output data (Season 1)	
**Cultivar**	**Season 1**	**Season 2**	**State**	**Sampling date (Season 1)**	**Season 1**	**Season 2**	**Million reads**	**% reads counted**
Desmayo Largueta	January 28th	January 27th	A	11/10/2015	0	5.0	106.2	73.6
Desmayo Largueta			A-B	12/1/2015	7.1	9.1	121.3	73.4
Desmayo Largueta			B	12/21/2015	16.1	20.1	108.3	74.5
Penta	February 10th	February 2nd	A	11/10/2015	0	5.0	91.9	76.8
Penta			A-B	1/12/2016	25	29.2	115	74.3
Penta			B	2/10/2016	41.2	53.8	96.9	74.6
Tardona	February 23rd	February 10th	A	11/10/2015	0	5.0	90	75.4
Tardona			A-B	2/10/2016	41.2	44.8	105.3	74.4
Tardona			B	3/1/2016	49.2	60.6	99.5	75.3
